# Reframing metformin as a gut-targeted glucose-lowering therapy: Mechanistic insights and translational relevance

**DOI:** 10.1016/j.molmet.2025.102276

**Published:** 2025-10-25

**Authors:** Tongzhi Wu, Michael Horowitz, Karen L. Jones, Christopher K. Rayner

**Affiliations:** 1 Adelaide Medical School and Centre of Research Excellence in Translating Nutritional Science to Good Health, The University of Adelaide, Adelaide, Australia; 2 Endocrine and Metabolic Unit, Royal Adelaide Hospital, Adelaide, Australia; 3 Department of Gastroenterology and Hepatology, Royal Adelaide Hospital, Adelaide, Australia

‘It’s tough to make predictions, especially about the future’ – Yogi Berra.

## 1 Introduction

The development and use of metformin – a longstanding cornerstone of type 2 diabetes mellitus (T2DM) therapy and a World Health Organisation essential medication – attest to the unpredictability of both scientific advancement and clinical translation. The quote from the legendary American baseball player and coach, Yogi Berra, well known for his malapropisms, is therefore apt. Originally derived from a plant (*Galega officinalis)* and used traditionally as a folk remedy, metformin’s substantial glucose-lowering capacity, without the induction of hypoglycaemia, was first appreciated in 1929. Despite this early recognition, metformin was not introduced for the management of T2DM until 1957 (in France) and only gained approval from the US Food and Drug Administration in 1995. It is now appreciated that metformin also has pleotropic properties beyond glucose lowering, which are being explored in diverse contexts, including ageing, cancer, and both cardiovascular and neurological disorders. Given its erratic development, it is perhaps not surprising that recent advances have redefined both the sites and mechanisms underlying the glucose-lowering action of metformin – a paradigm shift from the liver to the intestine [1]. This commentary summarises these insights and their substantial implications for refining the clinical application of metformin, particularly in settings where use of conventional formulations is constrained or contraindicated, such as gestational diabetes mellitus (GDM) and T2DM associated with renal impairment.

## 2 Sites and mechanisms of metformin action: from the liver to the gut

After oral administration, only about half of a metformin dose is absorbed in the small intestine and it accumulates in both the liver and the intestinal mucosa in concentrations that greatly exceed those in plasma – up to 10-fold and 300-fold, respectively [1]. These distinctive distribution patterns *per se* provide a strong rationale for clarifying the importance of hepatic and gastrointestinal actions to its glucose-lowering effect.

In a landmark study reported in 1995 [2], Stumvoll et al. demonstrated that in individuals with T2DM, metformin reduced hepatic glucose output by up to 75%, primarily through suppression of gluconeogenesis. This seminal observation established the longstanding ‘hepatic paradigm’ of metformin action and prompted intensive, and still ongoing, investigation to define the relevant hepatocellular mechanisms. Early studies demonstrated that metformin, in concentrations that often exceeded those achievable in clinical settings [3], inhibited mitochondrial complex I of the respiratory chain within hepatocytes to reduce ATP production, activate AMP-activated kinase (AMPK) and suppress gluconeogenesis [[4], [5], [6]]. Subsequent studies demonstrated that lower doses of metformin activate hepatic AMPK (e.g. intraperitoneal injection of 50 mg/kg/day in mice [7]). Interestingly, metformin also inhibits hepatic gluconeogenesis in AMPK knockout models [8], an observation which prompted investigation of potential AMPK-independent mechanisms. In this context, metformin was shown to suppress adenylate cyclase activity to attenuate glucagon-stimulated cyclic AMP production and downstream protein kinase A signalling [9]. Furthermore, metformin-induced inhibition of the mitochondrial glycerophosphate dehydrogenase (mGPD) may further limit gluconeogenic flux [10]. If the glucose-lowering effect of metformin was mediated exclusively within hepatocytes, its efficacy would be expected to be dependent on hepatic uptake via the organic cation transporter 1 (OCT1). However, in a large retrospective study, individuals with T2DM carrying loss-of-function variants in the OCT1-encoding gene (*SLC22A1*) exhibited comparable reductions in glycaemia to those with intact transporter function [11]. This finding indicates that hepatic mechanisms alone do not fully account for the glucose-lowering effect of metformin and that extra-hepatic actions are likely to be important.

The importance of ‘intestinal’ mechanisms in mediating glucose-lowering by metformin has become increasingly apparent. For example, in a rodent model of T2DM, metformin reduced elevated blood glucose much more effectively after enteral, than intravenous, administration [12]. In T2DM, the glucose-lowering capacity of delayed-release metformin – formulated to reside primarily in the gut with minimal systemic bioavailability – was shown to be comparable to that of conventional immediate- and extended-release formulations [13,14]. By contrast, in individuals with T2DM, a stepped intravenous infusion of metformin, leading to ‘therapeutic’ plasma concentrations, had little, if any, effect on either peripheral glucose disposal or hepatic glucose production during a hyperglycaemic clamp [15]. Together, these findings established metformin is also a gut-targeted glucose-lowering therapy. Indeed, there is increasing evidence that metformin exerts multiple gastrointestinal effects unrelated to hepatic function, including slowing of gastric emptying [16,17], enhancing secretion and/or signalling of the incretin hormone, glucagon-like peptide-1 (GLP-1) [[16], [17], [18], [19], [20]], inhibiting intestinal glucose absorption [[21], [22], [23], [24], [25]], and modulating bile acid signalling [26,27], the gut microbiota [28], and epithelial integrity [29]. Identifying which of these actions represent significant contributors to the glucose-lowering effect of metformin has important therapeutic implications.

In the current issue of *Molecular Medicine*, Godet et al. [30] addressed this question in a comprehensive, four-week dose–response study in high-fat, high-sucrose diet-fed mice. The effects of metformin (50–300 mg/kg/day) on microbiota composition, bile acids, ileal farnesoid X receptor (FXR) – fibroblast growth factor (FGF15) signalling, GLP-1, goblet cells, epithelial morphology, liver ceramides, and circulating metabolites, were evaluated. Predictably, glucose homeostasis improved in a dose-dependent manner, with maximal effects on insulin sensitivity and glucose tolerance at the highest doses of metformin. Reductions in FXR–FGF15 signalling and hepatic ceramide were evident even at low doses, consistent with threshold effects, while morphological changes in the intestinal epithelium and increases in the density of GLP-1-positive cells exhibited a dose-dependent response that paralleled improvements in glucose tolerance. Metformin also reshaped the gut microbiota to enrich beneficial taxa (e.g. *A. muciniphila*, *L. reuteri*, *L. johnsonii*) and suppress potentially harmful species, with heterogeneous dose–response relationships. Bile acids were observed to shift towards a higher FXR antagonist-to-agonist ratio, with increases in tauro-conjugates and reductions in sulfo-conjugates, although dose dependence was not evident. Plasma metabolomics further revealed normalisation of microbial metabolites linked to insulin resistance. While observational, these clear-cut findings align strongly with the outcomes of prior mechanistic studies, in which blockade of GLP-1 signalling by exendin(9–39) attenuated the glucose-lowering effect of metformin in T2DM [19] and transplantation of faeces from metformin-treated individuals with T2DM improved glucose tolerance in high-fat diet-fed mice [28]. Accordingly, these outcomes collectively indicate that gut-driven mechanisms, particularly the reinforcement of intestinal barriers, enhancement of the endogenous GLP-1 system, and changes in the microbiota, represent a substantial contributor to the capacity of metformin to lower elevated blood glucose levels. Studies are now required to quantify the relative contribution of these gut-mediated mechanisms in humans and clarify their interaction with hepatic actions to improve glycaemic control.

## 3 Translational implications

The recognition of metformin as a potentially gut-targeted glucose-lowering therapy has important implications for the refinement of its clinical application. When metformin was considered primarily to lower fasting hyperglycaemia by suppressing hepatic glucose output, standard advice was to administer it with meals to minimise the potential for gastrointestinal adverse effects (e.g. nausea and diarrhoea). However, a recent study in people with T2DM managed by metformin demonstrated that administration of metformin 30–60 min before a nutrient load, when compared to its administration with the nutrient load, stimulates GLP-1 secretion and lowers the subsequent glycaemic response more, apparently without increasing gastrointestinal adverse effects [18]. Larger and longer-term studies are required in T2DM to confirm the apparently greater efficacy and tolerability of pre-meal dosing. There is also evidence that metformin-induced slowing of gastric emptying and stimulation of GLP-1 secretion may help stabilise blood pressure after meals to mitigate the risk of postprandial hypotension – a fall in systolic blood pressure of ≥20 mmHg within 2 h of a meal – that is a common yet poorly appreciated condition, strongly associated with falls, syncope, stroke and increased mortality [16,31]. That the glucose-lowering efficacy of delayed-release metformin, relative to immediate- or extended-release formulations, is comparable further highlights the therapeutic potential of gut-targeted approaches [13,14]. By confining drug exposure to the gut, systemic absorption is minimised, or potentially avoided, which is desirable in T2DM associated with renal impairment. Dedicated trials in the latter would advance this concept. Similarly, limiting (or avoiding) systemic exposure may alleviate concerns regarding placental transfer and a potential adverse effect on foetal growth in women with GDM or T2DM with pregnancy, but these remain to be established. Finally, the dose-dependent microbial and epithelial alterations observed by Godet et al. [30] raise the possibility that these features serve as biomarkers for the therapeutic response to metformin and/or as targets for adjunctive interventions. Collectively, these important, emerging insights support the relevance of future research and potential clinical optimisation based on a ‘gut-centric’ perspective.

## 4 Conclusions

The outcomes of mechanistic and translational studies establish that the glucose-lowering effect of metformin in T2DM is mediated not only by suppression of hepatic glucose production, but also, and perhaps predominantly, through actions on the stomach and intestine. Recognition of these effects has reframed metformin as a ‘gut-targeted’ therapy in T2DM and stimulated novel strategies, such as pre-meal administration and gut-restricted formulations. Looking ahead, there are a number of priorities with the potential to advance the field significantly beyond current consensus. First, definition of gut-specific biomarkers (e.g. microbial, bile acid, and epithelial signatures) as predictors of both glucose lowering and gastrointestinal tolerability may lead to more personalised use of metformin. Second, elucidating the molecular pathways linking metformin to bile acid metabolism, FXR antagonism, and GLP-1 secretion may well reveal novel therapeutic targets with broader metabolic benefits. Finally, the development and testing of gut-restricted, or gut-selective metformin (or its mimetics) may extend its utility to individuals with renal impairment or pregnancy, where systemic exposure is undesirable. A ‘gut-centric’ perspective, accordingly, not only provides a more comprehensive understanding of the mode of action of metformin, but informs the refinement of its clinical application.

## CRediT authorship contribution statement

**Tongzhi Wu:** Writing – review & editing, Writing – original draft, Conceptualization. **Michael Horowitz:** Writing – review & editing, Conceptualization. **Karen L. Jones:** Writing – review & editing, Conceptualization. **Christopher K. Rayner:** Writing – review & editing, Conceptualization.

## Ethics approval and consent to participate

Not applicable.

## Consent for publication

Not applicable.

## Funding

The authors’ work in this field was supported by 10.13039/501100000971Diabetes Australia.

## Declaration of competing interest

The authors declare that they have no known competing financial interests or personal relationships that could have appeared to influence the work reported in this paper.

## Data Availability

No data was used for the research described in the article.

## List of abbreviations

T2DM: type 2 diabetes mellitus
GDM: gestational diabetes mellitus
GLP-1: glucagon-like peptide-1
mGPD: mitochondrial glycerophosphate dehydrogenase
AMPK: AMP-activated protein kinase
FXR: farnesoid X receptor
FGF-15: fibroblast growth factor

## References

[1] Wu T., Horowitz M., Rayner C.K. (2017). New insights into the anti-diabetic actions of metformin: from the liver to the gut. Expet Rev Gastroenterol Hepatol.

[2] Stumvoll M., Nurjhan N., Perriello G., Dailey G., Gerich J.E. (1995). Metabolic effects of metformin in non-insulin-dependent diabetes mellitus. N Engl J Med.

[3] He L., Wondisford F.E. (2015). Metformin action: concentrations matter. Cell Metab.

[4] Owen M.R., Doran E., Halestrap A.P. (2000). Evidence that metformin exerts its anti-diabetic effects through inhibition of complex 1 of the mitochondrial respiratory chain. Biochem J.

[5] El-Mir M.Y., Nogueira V., Fontaine E., Averet N., Rigoulet M., Leverve X. (2000). Dimethylbiguanide inhibits cell respiration via an indirect effect targeted on the respiratory chain complex I. J Biol Chem.

[6] Zhou G., Myers R., Li Y., Chen Y., Shen X., Fenyk-Melody J. (2001). Role of AMP-activated protein kinase in mechanism of metformin action. J Clin Investig.

[7] Zhang C.S., Li M., Ma T., Zong Y., Cui J., Feng J.W. (2016). Metformin activates AMPK through the lysosomal pathway. Cell Metab.

[8] Foretz M., Hebrard S., Leclerc J., Zarrinpashneh E., Soty M., Mithieux G. (2010). Metformin inhibits hepatic gluconeogenesis in mice independently of the LKB1/AMPK pathway via a decrease in hepatic energy state. J Clin Investig.

[9] Miller R.A., Chu Q., Xie J., Foretz M., Viollet B., Birnbaum M.J. (2013). Biguanides suppress hepatic glucagon signalling by decreasing production of cyclic AMP. Nature.

[10] Pryor R., Cabreiro F. (2015). Repurposing metformin: an old drug with new tricks in its binding pockets. Biochem J.

[11] Zhou K., Donnelly L.A., Kimber C.H., Donnan P.T., Doney A.S., Leese G. (2009). Reduced-function SLC22A1 polymorphisms encoding organic cation transporter 1 and glycemic response to metformin: a GoDARTS study. Diabetes.

[12] Stepensky D., Friedman M., Raz I., Hoffman A. (2002). Pharmacokinetic-pharmacodynamic analysis of the glucose-lowering effect of metformin in diabetic rats reveals first-pass pharmacodynamic effect. Drug Metab Dispos.

[13] DeFronzo R.A., Buse J.B., Kim T., Burns C., Skare S., Baron A. (2016). Once-daily delayed-release metformin lowers plasma glucose and enhances fasting and postprandial GLP-1 and PYY: results from two randomised trials. Diabetologia.

[14] Buse J.B., DeFronzo R.A., Rosenstock J., Kim T., Burns C., Skare S. (2016). The primary glucose-lowering effect of metformin resides in the gut, not the circulation: results from short-term pharmacokinetic and 12-Week dose-ranging studies. Diabetes Care.

[15] Sum C.F., Webster J.M., Johnson A.B., Catalano C., Cooper B.G., Taylor R. (1992). The effect of intravenous metformin on glucose metabolism during hyperglycaemia in type 2 diabetes. Diabet Med.

[16] Quast D.R., Xie C., Bound M.J., Grivell J., Hatzinikolas S., Jones K.L. (2025). Effects of metformin on postprandial blood pressure, heart rate, gastric emptying, GLP-1, and prevalence of postprandial hypotension in type 2 diabetes: a double-blind placebo-controlled crossover study. Diabetes.

[17] Borg M.J., Bound M., Grivell J., Sun Z., Jones K.L., Horowitz M. (2019). Comparative effects of proximal and distal small intestinal administration of metformin on plasma glucose and glucagon-like peptide-1, and gastric emptying after oral glucose, in type 2 diabetes. Diabetes Obes Metabol.

[18] Xie C., Iroga P., Bound M.J., Grivell J., Huang W., Jones K.L. (2024). Impact of the timing of metformin administration on glycaemic and glucagon-like peptide-1 responses to intraduodenal glucose infusion in type 2 diabetes: a double-blind, randomised, placebo-controlled, crossover study. Diabetologia.

[19] Bahne E., Sun E.W.L., Young R.L., Hansen M., Sonne D.P., Hansen J.S. (2018). Metformin-induced glucagon-like peptide-1 secretion contributes to the actions of metformin in type 2 diabetes. JCI Insight.

[20] Maida A., Lamont B.J., Cao X., Drucker D.J. (2011). Metformin regulates the incretin receptor axis via a pathway dependent on peroxisome proliferator-activated receptor-alpha in mice. Diabetologia.

[21] Ikeda T., Iwata K., Murakami H. (2000). Inhibitory effect of metformin on intestinal glucose absorption in the perfused rat intestine. Biochem Pharmacol.

[22] Wilcock C., Bailey C.J. (1991). Reconsideration of inhibitory effect of metformin on intestinal glucose absorption. J Pharm Pharmacol.

[23] Sakar Y., Meddah B., Faouzi M.A., Cherrah Y., Bado A., Ducroc R. (2010). Metformin-induced regulation of the intestinal D-glucose transporters. J Physiol Pharmacol : An Off J Polish Physiol Soc.

[24] Borg M.J., Xie C., Chen C., Bound M.J., Grivell J., Huang W. (2025). Metformin slows intestinal glucose absorption in type 2 diabetes, irrespective of the timing of its administration. Diabetes Obes Metabol.

[25] Wu T., Xie C., Wu H., Jones K.L., Horowitz M., Rayner C.K. (2017). Metformin reduces the rate of small intestinal glucose absorption in type 2 diabetes. Diabetes Obes Metabol.

[26] Sansome D.J., Xie C., Veedfald S., Horowitz M., Rayner C.K., Wu T. (2020). Mechanism of glucose-lowering by metformin in type 2 diabetes: role of bile acids. Diabetes Obes Metabol.

[27] Rohde U., Sonne D.P., Christensen M., Hansen M., Bronden A., Torang S. (2016). Cholecystokinin-induced gallbladder emptying and metformin elicit additive glucagon-like peptide-1 responses. J Clin Endocrinol Metab.

[28] Wu H., Esteve E., Tremaroli V., Khan M.T., Caesar R., Manneras-Holm L. (2017). Metformin alters the gut microbiome of individuals with treatment-naive type 2 diabetes, contributing to the therapeutic effects of the drug. Nat Med.

[29] Xue Y., Zhang H., Sun X., Zhu M.J. (2016). Metformin improves ileal epithelial barrier function in Interleukin-10 deficient mice. PLoS One.

[30] Godet M., Meugnier E., Vitalis O., Bendridi N., Vieille-Marchiset A., Vega N. (2025). Evaluation of the effects of metformin on gut functions and microbiota and their contribution to improving glucose tolerance in diabetic mice. Mol Metabol.

[31] Borg M.J., Jones K.L., Sun Z., Horowitz M., Rayner C.K., Wu T. (2019). Metformin attenuates the postprandial fall in blood pressure in type 2 diabetes. Diabetes Obes Metabol.

